# Engineered Biopolymeric Scaffolds for Chronic Wound Healing

**DOI:** 10.3389/fphys.2016.00341

**Published:** 2016-08-05

**Authors:** Laura E. Dickinson, Sharon Gerecht

**Affiliations:** ^1^Gemstone BiotherapeuticsBaltimore, MD, USA; ^2^Department of Chemical and Biomolecular Engineering, Institute for NanoBioTechnology, Johns Hopkins UniversityBaltimore, MD, USA

**Keywords:** chronic wounds, biopolymeric scaffolds, skin substitutes, acellular matrices, matrix remodeling, skin regeneration, inflammatory

## Abstract

Skin regeneration requires the coordinated integration of concomitant biological and molecular events in the extracellular wound environment during overlapping phases of inflammation, proliferation, and matrix remodeling. This process is highly efficient during normal wound healing. However, chronic wounds fail to progress through the ordered and reparative wound healing process and are unable to heal, requiring long-term treatment at high costs. There are many advanced skin substitutes, which mostly comprise bioactive dressings containing mammalian derived matrix components, and/or human cells, in clinical use. However, it is presently hypothesized that no treatment significantly outperforms the others. To address this unmet challenge, recent research has focused on developing innovative acellular biopolymeric scaffolds as more efficacious wound healing therapies. These biomaterial-based skin substitutes are precisely engineered and fine-tuned to recapitulate aspects of the wound healing milieu and target specific events in the wound healing cascade to facilitate complete skin repair with restored function and tissue integrity. This mini-review will provide a brief overview of chronic wound healing and current skin substitute treatment strategies while focusing on recent engineering approaches that regenerate skin using synthetic, biopolymeric scaffolds. We discuss key polymeric scaffold design criteria, including degradation, biocompatibility, and microstructure, and how they translate to inductive microenvironments that stimulate cell infiltration and vascularization to enhance chronic wound healing. As healthcare moves toward precision medicine-based strategies, the potential and therapeutic implications of synthetic, biopolymeric scaffolds as tunable treatment modalities for chronic wounds will be considered.

## Introduction

In the United States and other developed countries, aging populations coupled with escalating rates of diabetes, and obesity have significantly contributed to the increased prevalence of chronic wounds. Chronic wounds fail to progress through the systematic and reparative wound healing process and instead remain unhealed for >12 weeks (Shultz et al., 2003). Most chronic wounds can be classified into three major wound types [diabetic foot ulcers (DFUs), leg ulcers and pressure ulcers] based on their underlying pathogenesis, i.e., diabetes mellitus, venous deficiencies, arterial perfusion, or unrelieved pressure (local tissue hypoxia) (Mustoe et al., 2006) However, factors such as advanced age, poor nutrition, and immunosuppression plague the patient demographic suffering with non-healing, chronic wounds. These factors cause additional cellular and systemic stress that further contribute to wound chronicity and delay healing.

Chronic wounds are estimated to affect more than 6.5 million patients in the United States alone, and the annual healthcare burden associated with their treatment is estimated to be in excess of $25 billion (Sen et al., 2009). Not only are chronic wounds incredibly painful for patients, significantly diminishing their quality of life, but they also require long-term treatment at high costs. Despite these high costs, reported recurrence rates for chronic ulcers remain extremely high, ranging from 23 to 40% for pressure ulcers, 24–57% for most chronic venous ulcers, and upward of 60% for diabetic ulcers (Werdin et al., 2009). One reason for their recurrence is because chronic wounds do not progress through the stages of normal wound healing. Even with current treatment modalities, chronic wounds are unable to regenerate tissue of complete functional integrity. The current standard of care currently focuses on compression, infection control, debridement, and selecting an appropriate dressing that maintains a moist wound healing environment. Complete wound closure is the primary clinical outcome for chronic wounds, however, successful wound closure does not necessarily correlate to regenerated tissue of a higher quality, which is desired because it is more resistant to wound dehiscence and recurrence.

## Wound healing

Classic wound healing is a dynamic yet well-orchestrated and highly efficient process that requires the interaction of numerous cell types, soluble mediators, and the extracellular milieu to proceed linearly through the wound healing cascade: inflammation, re-epithelialization, angiogenesis, granulation tissue formation, wound contraction, and tissue maturation (Singer and Clark, 1999; Blakytny and Jude, 2006). During inflammation, aggregated platelets release growth factors, and pro-inflammatory chemokines to recruit neutrophils and macrophages to the local wound site. These inflammatory cell types phagocytose debris and bacteria and secrete mediators to stimulate the chemotaxis of cell types necessary for the proliferative phase. During the proliferative phase, fibroblasts, keratinocytes, endothelial and smooth muscle cells migrate through the wound, and proliferate to re-epithelialize the denuded surface, synthesize and deposit a provisional extracellular matrix, form new blood vessels, and contract the wound size. During the final stage, the newly formed granulation tissue is remodeled by the activity of matrix metalloproteinases (MMPs) balanced with tissue inhibitors of metalloproteinases (TIMPs), which rearranges the loose, regenerated dermis, and strengthens the repaired tissue (Gurtner et al., 2008).

Disruption of this normal wound healing cascade results in the development of non-healing chronic wounds. There is a perpetual antagonism between pro- and anti-inflammatory cytokines and an excess of oxygen free radicals and proteases, which creates a hostile microenvironment, and maintains chronic wounds in a prolonged state of inflammation that is unable to progress through later phases of wound healing. Indeed, chronic wounds display a myriad of cellular, and molecular abnormalities, many of which are attributed to dysregulated, and dysfunctional interactions between cellular constituents and the ECM (Schultz and Wysocki, 2009).

## Aberrant microenvironment of chronic wounds

The wound microenvironment presents a myriad of instructive biochemical cues, cell adhesive sites and molecules within a structural framework of essential matrix proteins—the ECM. The ECM provides structural support and tensile strength, attachment sites for cell surface receptors, and a reservoir for signaling factors that regulate cell migration, proliferation, and angiogenesis. The ECM has a complex 3D architecture of fibrous proteins, polysaccharides and proteoglycans that are secreted by fibroblast and epidermal cells, and it plays a significant and dynamic role in wound healing (Badylak, 2002; Tracy et al., 2016).

Classic wound healing is a cascade of overlapping events through bidirectional interaction between various cell types and the ECM. For instance, fibroblasts synthesize and secrete collagen and ECM components, which in turn concomitantly regulates fibroblast function, such as migration, collagen synthesis, and myofibroblast differentiation (Bainbridge, 2013). Chronic wounds exhibit a host of aberrant cellular and biochemical elements that contributes to their state of persistent inflammation and significantly impairs healing. Fibroblasts from chronic wounds are phenotypically different from those in acute wounds; they are senescent and exhibit diminished migration, reduced proliferation, and decreased collagen synthesis (Lerman et al., 2003). Coupled with inhibited ECM deposition is elevated protease activity, including upregulated and amplified activity of MMPs, collagenase, elastase, and serine proteases (Vaalamo et al., 1996; McCarty and Percival, 2013). The excess of proteases degrade fibrillar collagen I to non-bioactive gelatin, cleave signaling sequences from proteins, and inactivate growth factors (Eming et al., 2007). Indeed, fluid from chronic wounds, but not acute wounds, has been found to rapidly degrade platelet derived growth factor (PDGF), transforming growth factor (TGF-β1), and angiogenic vascular endothelial growth factor (VEGF) (Lauer et al., 2000). The excessive degradation of the ECM, proteins and growth factors deprives cells of attachment sites and vital signals, subsequently disrupting the progression of wound healing (Shultz et al., 2012).

Keratinocytes are also dysfunctional in chronic wounds. In normal wound repair, keratinocytes migrate as a cell sheet over the granulation tissue, and differentiate to re-epithelialize the skin via integrin mediated binding interactions with ECM molecules (Santoro and Gaudino, 2005). However, in chronic wounds, although keratinocytes are hyperproliferative, they are unable to migrate and close the wound (Pastar et al., 2008). This poor migratory ability is concomitantly attributed to altered integrin expression (Hakkinen et al., 2004) and the degraded ECM components. Instead, keratinocytes at the non-healing edges of chronic wounds continuously proliferate, forming a thick, hyperkeratotic layer. Contributing to poor epithelialization is the overall excessive inflammatory tissue microenvironment, which inhibits the migration of fibroblasts and the synthesis of new ECM, and the loss of epidermal stem cell (ESC) populations. ESC populations reside in distinct compartments or niches that regulate their self-renewal and lineage fate (Braun and Prowse, 2006); in response to tissue injury, the ESCs proliferate, differentiate, and migrate to re-epithelialize the wounded area (Cha and Falanga, 2007). However, in chronic venous ulcers, it has been shown that there is a loss of SC niche signaling and subsequent deregulation and depletion of ESCs that possibly contributes to the hyperpoliferative epidermis of a non-healing venous ulcer wound edge (Stojadinovic et al., 2014).

Although far from an exhaustive summary, the discussion above emphasizes the biological complexity of chronic wounds. Indeed, the impairment of the ECM in chronic wounds has long been identified as a key target for wound healing strategies. Within the last 20 years, substantial emphasis has been directed toward the development of bioengineered skin substitutes, such as living skin equivalents, acellular matrices, and polymeric scaffolds, that recapitulate multiple features of the native ECM that are so necessary in regulating the wound healing cascade (Rennert et al., 2013). Several bioengineered scaffolds that are FDA approved and commercially available will be discussed in this review. All of the wound healing skin substitutes discussed in this mini-review provide an ECM, whether natural or synthetic, that supports the infiltration of cells, tissue regeneration, and ultimately wound closure. These skin substitutes are designed to provide a bio-inductive and vulnerary environment by modulating the proteolytic climate and/or supplementing the wound bed with exogenous, bioactive factors that stimulate innate tissue repair mechanisms. However, to date, there have been limited head-to-head comparative clinical studies evaluating the performance of the plethora of advanced wound care products, which are required to guide clinical practice and payer determinations (Valle et al., 2014).

## Bioengineered skin substitutes

The optimal bioengineered scaffold for skin regeneration of chronic wounds should (1) be non-immunogenic; (2) modulate proteolytic activity to reset the wound to an acute state; (3) provide a bio-resorbable scaffold that facilitates cellular migration and promotes cellular proliferation and matrix deposition; (4) recruit angiogenic and fibroblast cell types to synthesize granulation tissue; and (5) absorb and neutralize free radicals (Gould, 2015). In the following sections, we will describe the various types of bioengineered skin substitutes, including those that contain natural ECM components harvested from human tissue or animal sources and synthesized, ECM-mimetic biopolymeric scaffolds. All scaffolds detailed in this review are listed in Table 1.

**Table 1:** **Summary of scaffolds for chronic wound healing**.

**Product**	**Composition**	**Properties/Mechanism of action**	**FDA**
**LIVING SKIN SUBSTITUTES**			
Apligraf®	Bovine type I collagen seeded with human neonatal fibroblasts and keratinocytes	Metabolically active cells secrete cytokines and growth factors to stimulate differentiation and proliferation	PMA (1998)
Dermagraft®	Human neonatal fibroblasts seeded on bioabsorbable polyglactin scaffold—cryopreserved	Metabolically active fibroblasts secrete collagen, matrix proteins, growth factors and cytokines	PMA (2001)
TheraSkin®	Cryopreserved skin allograft harvested from cadavers	Biologically active scaffold providing cellular and extracellular components Natural barrier to infection	HCT/Ps
**ACELLULAR NATURALLY DERIVED POLYMERIC SCAFFOLDS**			
Oasis®	Minimally processed ECM derived from porcine small-intestine submucosa	Provides structural matrix and delivers growth factors to stimulate angiogenesis and cell migration	510 K (1998)
GraftJacket®	Processed (crosslinked and cryopreserved) human dermal matrix	Fenestrated acellular matrix that acts as a foundation for revascularization and cellular repopulation Reduces inflammation	HCT/Ps
DermACELL®	Decellularized human dermis allograft	Unique anionic detergent and endonuclease-based process to decellularize tissue Scaffold supports cell ingrowth	HCT/Ps
EpiFix®	Dehydrated allograft: amnion and chorion membranes derived from donated human placenta	Composed of a single layer of epithelial cells, a basement membrane and an avascular connective tissue matrix Retains soluble biological molecules and growth factors that stimulate human dermal fibroblast proliferation and the migration of human mesenchymal stem cells	HCT/Ps
Integra™	Cross-linked bovine collagen and chondroitin 6-sulfate with a silicone membrane	Biodegradable matrix provides a scaffold for cellular invasion and capillary growth	PMA (1996) 510 K (2002)
Promogran™	Freeze-dried composite of 55% collagen and 45% oxidized regenerated cellulose	Composite matrix absorbs wound exudate to form a biodegradable gel Provides a scaffold for cellular migration	510 K (2002)
Tegagen™, Algisite™, Algi-Fiber, etc.	Dressings of calcium alginate fibers	Form gelatinous mass upon contact with wound exudate Extremely absorbent (10 ×) Controls mild hemorrhages	510 K
**BIOPOLYMERIC SCAFFOLDS**			
Talymed®	Shortened fibers of N-acetyl glucosamine isolated from microalgae	Material interacts with fibroblasts and endothelial cells to stimulate cell migration	510 K (2010)
Hyalomatrix®	Non-woven pad of benzyl ester of hyaluronic acid and a semipermeable silicone membrane	Biodegradable scaffold for cellular invasion and capillary growth. Contains a semipermeable silicone membrane to prevent water loss	510 K (2007)
Dextran	Crosslinked modified dextran and PEG diacrylate	Biodegradable matrix fills wound defect and provides a scaffold for cellular infiltration	N/A

*Pre-market approval (PMA); Human cells, tissues, or cellular-based products (HCT/Ps)*.

### Living skin equivalents: human-derived technologies

Living skin equivalents comprise scaffolds, either natural, or synthetic, seeded with allogenic fibroblasts, and/or keratinocytes. There are several iterations of products that have been developed using this approach, which essentially provide cellular, and structural components for wound healing. Apligraf® (Organogenesis, Inc.) is composed of a bovine type I collagen matrix seeded with neonatal fibroblasts to produce a neodermal layer. Human neonatal epidermal keratinocytes are subsequently added on top of this dermal component as a monolayer to approximate the epidermis and form a differentiated stratum corneum (Zaulyanov and Kirsner, 2007). This results in a metabolically active bilayered skin substitute providing both a dermal and epidermal layer with living cells. Although the fibroblasts and keratinocytes in Apligraf do not persist beyond 6 weeks in patients (Hu et al., 2006), they are thought to be responsible for stimulating differentiation and proliferation via secretion of essential cytokines and growth factors (Falanga et al., 2002). Apligraf was the first allogeneic cell-based product to be approved by the FDA in 1998 for the treatment of DFUs and venous leg ulcers. Large multicenter randomized clinical trials demonstrated a significantly higher rate of wound closure compared with conventional standard of care (Veves et al., 2001; Edmonds and European and Australian Apligraf Diabetic Foot Ulcer Study Group, 2009).

Dermagraft® (Organogenesis, Inc.) was approved by the FDA in 2001 for the treatment of non-healing DFUs. Although it also contains neonatal dermal fibroblasts, it differs from Apligraf in that the fibroblasts are cultured onto a bioresorbable polyglactin mesh scaffold; polyglactin is a standard suture material. The metabolically active fibroblasts proliferate within the interstices of the synthetic scaffold, secreting collagens, growth factors, cytokines, proteoglycans, and other key regulatory molecules, to create a 3-D bioactive matrix, which is then cryopreserved for storage (Naughton et al., 1997). When applied to DFUs, Dermagraft significantly increased the rate of wound closure compared to the control (Marston et al., 2003).

Another biologically active human skin allograft is TheraSkin® (Soluble Systems), which is harvested within 24 h post-mortem and cryogenically processed to preserve the viable fibroblasts, keratinocytes, and fully developed ECM sequestered with essential growth factors and cytokines. It has been reported that Theraskin, which was found to be effective in the treatment of DFUs and venous stasis ulcers (Landsman et al., 2011), contains a greater quantity of the key collagens critical to wound healing compared to Apligraf (DiDomenico et al., 2011). This may be attributed to the manufacturing process of Apligraf in which a bovine collagen substrate is used to culture neonatal cells that deposit the ECM *in vitro*. The application of a living human dermal skin substitute delivers a smorgasbord of vital key regulatory proteins and cytokines that stimulate angiogenesis, fibroblast migration, and keratinocyte proliferation to accelerate wound healing. However, there is an absence of head-to-head studies that compare the clinical and cost efficacy advanced wound care products to inform clinical practice and payer determination. Indeed this stems also from the variety of chronic wound types with various etiologies—there is no single wound care product to treat and manage all wound types. Most comparative studies are either retrospective analyses or funded by the company. In a retrospective study evaluating the efficacy of EpiFix compared to Apligraf in treating DFUs, it was reported that patients treated with EpiFix required more applications compared to patients treated with Apligraf, and that the median time to wound closure using Apligraf was 13.3 weeks compared to 26 weeks for EpiFix (Kirsner et al., 2015b). However, in a prospective study, 97% of lower extremity diabetic ulcers healed when treated with EpiFix compared to only 73% of wounds treated with Apligraf, suggesting that patients treated with EpiFix experienced a shorter time to wound closure (Zelen et al., 2016). The median graft cost was $8,918 (range $1,486–19,323) per healed wound for the Apligraf group and $1517 (range $434–25,710) per healed wound in the EpiFix group (Zelen et al., 2016). In a separate retrospective analysis, treatment using the bilayered living cell construct Apligraf reduced the median time to wound closure of venous leg ulcers by 44% compared to treatment using a naturally derived, acellular porcine dressing (Oasis®; to be discussed below) (Kirsner et al., 2015b).

### Acellular naturally derived polymeric scaffolds

Acellular matrices are characterized as nonviable biomaterials. They may be animal- or human-derived, with all cells removed during manufacture, or they may be synthetic or a composite, where cells are simply not present from the outset. Natural polymers are commonly utilized in the development of acellular matrices for chronic wound treatments because of their inherent biocompatibility and bioactivity as well as their ability to mimic the structural, biomechanical, and biochemical functions of the ECM. There are cost advantages to using naturally derived ECM-based polymeric scaffolds. Using a Markov model to estimate the comparative cost effectiveness of Apligraf, Dermagraft, and an ECM-based therapy, the ECM-based therapy was economically dominant and determined to be the most cost effective for the management of venous leg ulcers as an adjunct therapy to standard of care (Carter et al., 2014). The expected costs for a naturally derived ECM-based scaffold (Oasis®), Apligraf and Dermagraft were $6732, $10, 638, and $11, 237, for 31, 29, and 17 ulcer free weeks, respectively, suggesting that naturally derived ECM-based therapies yield potential savings compared to other cell or tissue-derived products (Carter et al., 2014). The most common, bioactive natural polymers utilized in the development of acellular matrices for wound healing are collagen, hyaluronic acid, chitosan, and alginate.

Collagens are the most abundant ECM macromolecule and are the main component in human skin that provides structural integrity (Singer and Clark, 1999). In addition to its structural function, collagen I governs many cellular functions of fibroblasts and keratinocytes, including cell adhesion, differentiation, migration, ECM deposition, and angiogenesis (Heino, 2000; Gelse et al., 2003; Whelan and Senger, 2003). Collagen I is also able to bind excess proteases, inflammatory cytokines, and free radicals that are rampant in the chronic wound bed (Wiegand et al., 2010). The role of collagen in tissue repair and wound healing are multifactorial, which supports the extensive use of exogenous collagen-based scaffolds for chronic wound applications. Generally speaking, collagen-based scaffolds are classified as either decellularized matrices, derived from a variety of mammalian sources, and anatomical locations, including porcine small-intestine submucosa, or urinary bladder matrix, human cadavers, placental tissue, or they are synthesized via extraction and chemical crosslinking. There are many products that are currently used in the treatment of chronic wounds and several of them are briefly described below as representative examples.

Oasis® Wound Matrix (Healthpoint) is a naturally occurring ECM graft (>90% collagen) derived from porcine SIS, which is a thin, approximately 0.15 mm thick translucent layer of porcine intestine that is predominately type 1 collagen. Porcine SIS possesses a porous microstructure, with pores ranging in size from 20–30 μm that enables oxygen diffusion and promotes cell viability (Nihsen et al., 2008). Porcine SIS also retains the active forms of other biologically relevant components that provide cell and growth factor binding sites, sequester matrix-degrading enzymes, and enhance cellular infiltration into injured tissue. It is also embedded with glycosaminoglycans, proteoglycans, fibronectin, and various growth factors that imparts significant bioactivity (Hodde et al., 1996, 2001; Shi and Ronfard, 2013). In this way, the SIS not only provides a structural matrix and delivers growth factors to stimulate angiogenesis and cell migration but also regulates proteolytic activity and dampens the inhibitory effects of MMP-1, MMP-2, and MMP-9 on keratinocyte migration (Shi et al., 2012).

There are myriad acellular wound matrices available for clinical use that are processed, decellularized dermal constructs derived from donated human tissue. They are all designed to provide a scaffold for wound repair, however, each acellular dermal wound matrix differs by the way in which it is processed. For GraftJacket® (Wright Medical Technology), donated human tissue is treated to remove the epidermis and cellular components, but it retains collagen, elastin, proteoglycans, and the internal matrix of the dermis, which remains intact and is chemically crosslinked to maintain the collagen architecture before cryopreservation (Turner and Badylak, 2015). DermACELL® (LifeNet Health) is a human tissue matrix allograft that employs a unique, proprietary MATRACELL® technology (Moore et al., 2015) that uses anionic detergents and an endonuclease to achieve >97% nucleic acid removal while retaining biomechanical strength. This allows DermACELL to be preserved at ambient temperature and offer a >3 year shelf-life. Both of these products have been indicated for the treatment of non-healing ulcers and dermal wounds and have demonstrated the ability to reduce time to complete wound closure and increase healing rates compared to conventional care (Reyzelman et al., 2009; Yonehiro et al., 2013; Walters et al., 2016). In these processed acellular dermal matrices, the removal of the cellular components reduces the risk of rejection, and the critical dermal proteins that remain minimize inflammation and facilitate cell infiltration and tissue revascularization. In contrast to xenogeneic ECM allografts, minimally manipulated human tissue products are classified as human cell, tissues, and cellular and tissue-based products (HCT/Ps) by the FDA. As a result, there are fewer restrictions on the applications for which these devices can be used; they are viewed as tissue transplants and manufacturers are only required to follow manipulation guidelines to ensure materials are free from transmissible pathogens (Table 2).

**Table 2:** **Brief overview of United States FDA pathways for wound healing products (medical devices)**.

**Device classification/Risk**	**PHS 361: HCT/Ps low**	**510 K Class II/moderate**	**PMA Class III/high**
Review standard	No pre-market review Not required to demonstrate safety or effectiveness	Substantial equivalence in safety and effectiveness to a legally marketed predicate device	Approval requires that the safety and effectiveness of the device be established with valid scientific evidence, i.e., high-quality clinical data
Requirements	Minimally manipulated Intended for homologous use No systemic effect/not dependent on metabolic activity of cells Manufacturers follow good tissue practice to prevent the introduction, transmission, and spread of communicable diseases	Non-clinical laboratory studies for safety (performed under GLP conditions) *Clinical investigations not typically required* *Quality Systems in place prior to interstate commerce* *Manufacturing not reviewed pre-approval*	Non-clinical laboratory studies for safety (performed under GLP conditions) Clinical investigations (such as performed under an Investigational Device Exemption) Detailed Quality Systems in place Pre-approval of manufacturing facility with inspection
**Regulatory burden**	**Low**	**Medium**	**High**
Wound healing products	TheraSkin® GraftJacket® DermACELL® EpiFix®	Oasis® Integra®^*^ Promogran™ Tegagen™, Algisite™ Algi-Fiber Talymed® Hyalomatrix®	Apligraf® Dermagraft® Integra™^*^

*Pre-market approval (PMA); Human cells, tissues, or cellular-based products (HCT/Ps)*.

* *Although initial PMA was received in 1996, in April 2001, the FDA approved Integra™ Dermal Regeneration Template for marketing in the treatment of life-threatening full-thickness and/or deep partial thickness thermal injuries. Because the treatment of thermal injuries poses a significant risk, medical devices developed to treat burns are considered Class III devices that support of sustain human life. These products require a PMA submission accompanied with clinical data demonstrating safety and effectiveness. A separate 510(k) submission was filed for Integra™ Bilayer Matrix Wound Dressing, which was cleared for marketing in August 2002 for the management of a broad range of wound types, including partial, and full-thickness chronic wounds, surgical wounds (donor sites/grafts, post-Mohs surgery, post-laser surgery, podiatric, wound dehiscence), trauma wounds (abrasions, lacerations, second-degree burns, and skin tears) and draining wounds. Both Integra LifeSciences products are composed of cross-linked bovine tendon collagen with glycosaminoglycan and a semi-permeable polysiloxane membrane. With the 510(k) clearance, the Bilayer Matrix Wound Dressing is marketed to manage a broad range of wound indications, whereas the PMA limits indications to thermal injuries. However, as recently as January 2016, the Integra™ Dermal Regeneration Template was approved for indications including partial and full thickness neuropathic DFUs based on submitted clinical data*.

*www.FDA.gov*.

The use of placental membranes for wound healing has been reported for over 100 years, which can be attributed to its collagen-rich ECM presenting biologically active components, such as developmental cytokines and elevated concentrations of regenerative growth factors (Silini et al., 2015). Placental membranes contain a plethora of multifunctional growth factors, including, but not limited to, epidermal growth factor, basic fibroblast growth factor, PDGF, VEGF, TGF-β1, and keratinocyte growth factor, as well as MMPs and TIMPs (Fortunato et al., 1998; Koizumi et al., 2000; Lopez-Valladares et al., 2010) that support critical cell behavior and wound healing events. Also expressed in placental membranes are immunosuppressive factors and antibacterial peptides that contribute to the reduced risk of rejection of placental membranes (Park et al., 2008; Mamede et al., 2010). Large amounts of the ECM glycosaminoglycan hyaluronan (HA) are also present in placental membranes, which has been shown to function as a free radical scavenger to remove reactive oxygen species (Trabucchi et al., 2002; Lockington et al., 2014). However, different processing methods impact the composition and functionality of these materials (von Versen-Hoeynck et al., 2008). There are more than 25 commercially available placental membrane products, yet most contain no viable cells because they are either dehydrated or are cryopreserved devitalized or decellularized tissue. One such product, EpiFix® (MiMedx), is a dehydrated human tissue allograft comprising laminated amnion and chorion membranes derived from donated human placenta. During processing, the amnion and chorion tissue layers are isolated from the placenta and washed. The two layers are then laminated to form the graft, which is subsequently dehydrated and sterilized. EpiFix contains a single layer of epithelial cells, a basement membrane, and an avascular connective tissue matrix. After processing, EpiFix retains soluble biological molecules and growth factors that stimulate human dermal fibroblast proliferation and the migration of human mesenchymal stem cells (Koob et al., 2013). When evaluated in the treatment of DFUs and venous leg ulcers, EpiFix promoted complete epithelialization and reduced the wound size in patients compared to standard treatment (Zelen et al., 2013).

Integra™ (Integra Life Sciences) and Promogran™ (Systagenix Wound Management) are two wound healing products synthesized using extracted and polymerized collagen. Integra is a bilayer composite matrix of crosslinked collagen type I from bovine sources and a glycosaminoglycan (chondroitin 6-sulfate) isolated from shark skin. It has a semipermeable silicone membrane that functions as a temporary epidermal layer by controlling water vapor loss and providing structural integrity. The bilayer matrix recruits dermal fibroblasts to the wound, which then synthesize, and secrete new ECM to the wound bed to facilitate healing (Burke et al., 1982). Although initially indicated for third degree burns via an FDA pre-market approval (PMA; to be discussed below), a 300 subject clinical trial demonstrated that the non-healing DFUs treated with Integra had a more rapid time to complete wound closure and increased rate of wound closure compared to standard of care treatment (Driver et al., 2015). Promogran is a combination matrix composed of 55% bovine type I collagen and 45% oxidized regenerated cellulose (ORC) that is freeze dried and formed into a 3 mm thick sheet that is applied directly to the wound bed. Upon application, the composite matrix absorbs wound exudate to form a biodegradable gel that enhances fibroblast migration and proliferation (Hart et al., 2002). The composite matrix binds and stabilizes growth factors and physically sequesters and inactivates excessive MMPs while providing a scaffold for cellular migration (Cullen et al., 2002). Clinical studies demonstrate a significant reduction in the concentration and activity of proteases in the wound exudate of DFUs treated with Promogran and a greater reduction in wound size (Ulrich et al., 2011).

Other natural polymers used as wound dressings are alginate and chitosan. Alginate is a polysaccharide with homopolymeric blocks of 1,4-linked β-D-mannuronic and α-L-guluronic residues that is isolated from the cell walls of a variety of species of brown seaweed. Alginate exhibits unique gelation properties and ionically crosslinks in the presence of divalent ions to form a biocompatible 3D polymeric crosslinked scaffold for tissue engineering applications (Augst et al., 2006). Alginate wound dressings are used in wound management because they provide a moist environment, are highly absorbent, and function as a hemostat. When an alginate dressing comes into contact with wound exudate there is an ion exchange between the calcium ions in the mannuronic and gluronic groups of the alginate dressing and the sodium ions in blood or exudate (Segal et al., 1998). As sufficient calcium ions are replaced by sodium ions, the alginate fibers swell, partially dissolve, and form a gel that maintains a moist environment for autolytic debridement and reduces pain during dressing changes (Pirone et al., 1992). Alginate dressings have also been shown to minimize microbial bioburden and sequester proteinases (Sweeney et al., 2012). There are numerous alginate-based wound dressings approved for use in managing variety of wound types in which exudate is present, such as chronic wounds, including Tegagen™ (3M), Algisite™ (Smith and Nephew), and Algi-Fiber (CoreLeader Biotech) to name a few (Dumville et al., 2015; O'Meara et al., 2015).

Chitosan is a linear polysaccharide composed of randomly distributed β-(1–4)-linked D-glucosamine and N-acetyl-D-glucosamine that is predominately used as a hemostat but is currently being evaluated as a wound dressing for chronic wounds because of its ability to modulate the wound environment (Sandoval et al., 2011; Mayol et al., 2014). Chitosan is prepared by deacetylating chitin, the principle component in the exoskeleton of crustaceans, via enzymatic or alkaline hydrolysis before being processed into various fibrous, scaffold, and hydrogel biomaterials (Azuma et al., 2015). Chitosan contributes to wound healing by stimulating the rapid mobilization, adhesion, and aggregation of platelets and red blood cells to the wound site to facilitate rapid clotting (Chou et al., 2003; Okamotoa et al., 2003). Post-hemostasis, chitosan also accelerates granulation tissue and matrix formation (Biagini et al., 1992; Ueno et al., 2001). Lysozymes gradually depolymerize chitosan via hydrolysis to release N-acetyl-D-glucosamine, which stimulates fibroblast proliferation and collagen deposition and remodeling (Kojima et al., 2004). Chitosan has also been shown to stimulate inflammatory cells migration (Peluso et al., 1994). As a wound dressing biomaterial, chitosan exhibits several unique advantages, including non-toxicity, physiological inertness, antibacterial properties, biocompatibility, and an affinity to proteins (Dai et al., 2012). Chitosan's antimicrobial properties are attributed to the presence of primary amine groups that confer an overall cationic charge, which destabilizes, and permeabilizes microbial membranes (Rabea et al., 2003).

### Acellular matrices-biosynthetic polymeric scaffolds

Acellular synthetic matrices offer several advantages over naturally derived polymeric and cellular based scaffolds, including longer shelf-life, cost efficacy, and limited risk of rejection. In a retrospective study, the number of applications needed to treat (NNT) was used to model the comparative clinical and cost efficacy of currently available advanced wound care matrices as adjuncts to compression therapy for the treatment of venous leg ulcers. It was found that fewer applications of an acellular biosynthetic scaffold was required to achieve closure compared to a human skin equivalent (Apligraf) and biologically derived polymeric scaffold (Oasis) at a significantly lower cost. The incremental costs per additional successfully treated patient were $1600 for the acellular biosynthetic scaffold (Talymed®), $3150 for Oasis, and $29,952 for Apligraf (Hankin et al., 2012).

To design an efficacious biosynthetic polymeric scaffold that achieves wound closure and skin regeneration, several parameters, and criteria need to be considered in addition to those listed in the above section. Scaffolds that are chemically synthetized or modified not only need to be easily manufacturable but also biocompatible, biodegradable, and non-toxic while exhibiting optimal biomechanical properties, including ideal porosity, and morphology to modulate the transport cells, metabolites, and signaling molecules. Most importantly, cells must be able to appropriately respond to and infiltrate the scaffold to facilitate degradation and support a regenerative healing process (Kirsner et al., 2015a). Therefore, the fundamental design strategy in developing biosynthetic scaffolds is to recapitulate structural and molecular aspects of the ECM using tunable polymeric materials that simulate the elasticity and porosity of dermis. Polysaccharides possess reactive functional groups that can be modified to form non-toxic and bioactive wound healing biomaterials with optimized and tailorable characteristics, such as pore size and degradation rate, which elicit the appropriate biological response, and stimulate tissue regeneration. To date, there are few FDA cleared, commercially available biosynthetic scaffolds indicated for the use in managing chronic wounds. Two FDA cleared scaffolds, both of which are polysaccharide-based, will be discussed below. A brief overview of FDA device classification for wound healing scaffolds is also provided.

The United States FDA predominately regulates wound healing products as medical devices based on their composition and device classification, which depends on the intended use of the device. Devices that present relatively low risk are generally categorized as Class I or Class II devices, and higher risk devices are Class III. Minimally manipulated human-derived products, such as placental membrane-derived products, are regulated as human cells, tissues, and cellular and tissue-based products (HCT/Ps) and only require manufacturers to follow good tissue practices and manipulation guidelines. Class III human/animal-derived products, such as cellular wound matrices, are approved through a pre-market approval (PMA). Devices that present relatively low or moderate risk (Class II), such as animal-derived and synthetic products, require the manufacturer to seek 510(k) clearance, which is generally granted when submitted information establishes that a new device is “substantially equivalent” to an already approved and legally marketed “predicate” device in terms of technological characteristics, such as design, mode of action and composition, and performance. Many biosynthetic scaffolds are cleared through the 510(k) pathway (Table 2). In the European Union there are directives that outline requirements under which a medical device could be marketed across all E.U. member states after earning a Conformité Européenne (CE) mark in any one member country. These directives similarly categorize devices into four classes (I, IIa, IIb, and III) on the basis of associated risks. Approval and CE marks for medical devices are directly managed by designated Notified Bodies and are subject to performance and reliability testing. Approval is generally granted if the device successfully performs as intended in a manner in which the benefits outweigh expected risks. The specific requirements for pre-marketing clinical studies are vague, and the guidelines for the nature of these studies are not binding on manufacturers or Notified Bodies (Kramer et al., 2012).

Talymed® (Marine Polymer Technologies) is a biodegradable, wafer-thin wound matrix that was cleared in 2010 for the management of full and partial-thickness wounds, including chronic wounds. Talymed is a bioactive scaffold composed of shortened fibers of poly-N-acetyl glucosamine derived from diatom algae. Native poly-N-acetyl glucosamine fibers are shortened to ~4–7 μm using gamma radiation, which retains the unique 3D polymeric structure and enables the nanofibers to form a thin, biodegradable scaffold membrane (Scherer et al., 2009). Pre-clinical animal studies demonstrated that a nanofibrous scaffold composed of shortened poly-N-acetyl glucosamine fibers initiated wound healing through material-facilitated interactions with fibroblast and endothelial cells that stimulated re-epithelization via increased keratinocyte migration, granulation tissue formation, cell proliferation and vascularization (Scherer et al., 2009). The shortened fibers of poly-N-acetyl glucosamine become completely integrated into the wound bed and upregulate the integrin-dependent Ets1 transcription factor, which regulates genes involved in cell migration, proliferation and survival. The shortened fibers of poly-N-acetyl glucosamine stimulate endothelial cells and the increased secretion of several cytokines and growth factors, including IL-1 and VEGF, that are imperative for proper wound healing (Vournakis et al., 2008). In a pilot study, 86% of patients with venous leg ulcers that were treated with Talymed biweekly achieved complete wound healing within 5 months compared to patients only receiving standard of care (45%) (Kelechi et al., 2012).

Hyaluronan or hyaluronic acid (HA) is a linear glycosaminoglycan composed of alternating units of D-glucuronic acid and D-*N*-acetyl-d-glucosamine that is ubiquitously distributed within the ECM and specifically in connective tissue. HA is a well-established co-regulator for gene expression, proliferation, motility, adhesion, signaling, and morphogenesis (Toole, 2004). In wound healing, HA plays a key role in modulating inflammation, stimulating cell migration, and promoting angiogenesis through interactions with 2 cellular receptors: RHAMM and CD44 (Chen and Abatangelo, 1999). However, the role of HA in tissue repair is largely dependent on molecular size (Litwiniuk et al., 2016). High molecular weight HA exhibits anti-inflammatory, immunosuppressive, and anti-angiogenic effects by inhibiting EC proliferation, migration, and capillary formation, whereas short chain, low molecular weight degradation products of HA, namely oligosaccharides of 3–10 disaccharide units, are potent pro-inflammatory molecules that induce angiogenesis by stimulating EC proliferation, migration, and angiogenic sprouting (West and Fan, 2002). *In vivo*, native HA is subject to rapid enzymatic degradation by hyaluronidases and, in wounded tissue, further fragmentation by free radicals (Stern, 2004). Fortunately, HA is amenable to chemical modifications due to the presence of carboxyl and hydroxyl groups on its repeating disaccharide units. The functional groups allow HA-based biomaterials to be tailored to retard and control degradation for tissue regeneration and wound healing applications. Indeed, synthetic HA derivatives have been chemically modified by esterification of the carboxylic group of glucuronic acid with benzyl groups (Benedetti et al., 1993). This modification imparts higher resistance to hyaluronidase enzymatic activity and degradation. Hyalomatrix® (Anika Therapeutics) is a bilayered wound device composed of a wound contact layer containing fibers of esterified HA and an outer semipermeable silicone membrane that acts as a barrier to prevent vapor loss and reduce bacterial colonization. Hyalomatrix acts as a regenerative matrix by providing HA in the form of a 3D scaffold. The scaffold enables rapid fibroblast and endothelial cell infiltration and modulates ECM deposition (Galassi et al., 2000; Turner et al., 2004). In slow-healing wounds, as the matrix degrades, a high concentration of HA is locally released to the wound site that stimulates a regenerative response. When evaluated in wounds of different etiologies, including vascular, DFUs, traumatic wounds, and pressure ulcers, 83% of Hyalomatrix treated wounds achieved some degree of re-epithelialization (≥10%) within 16 days (Caravaggi et al., 2011).

**Figure 1:**
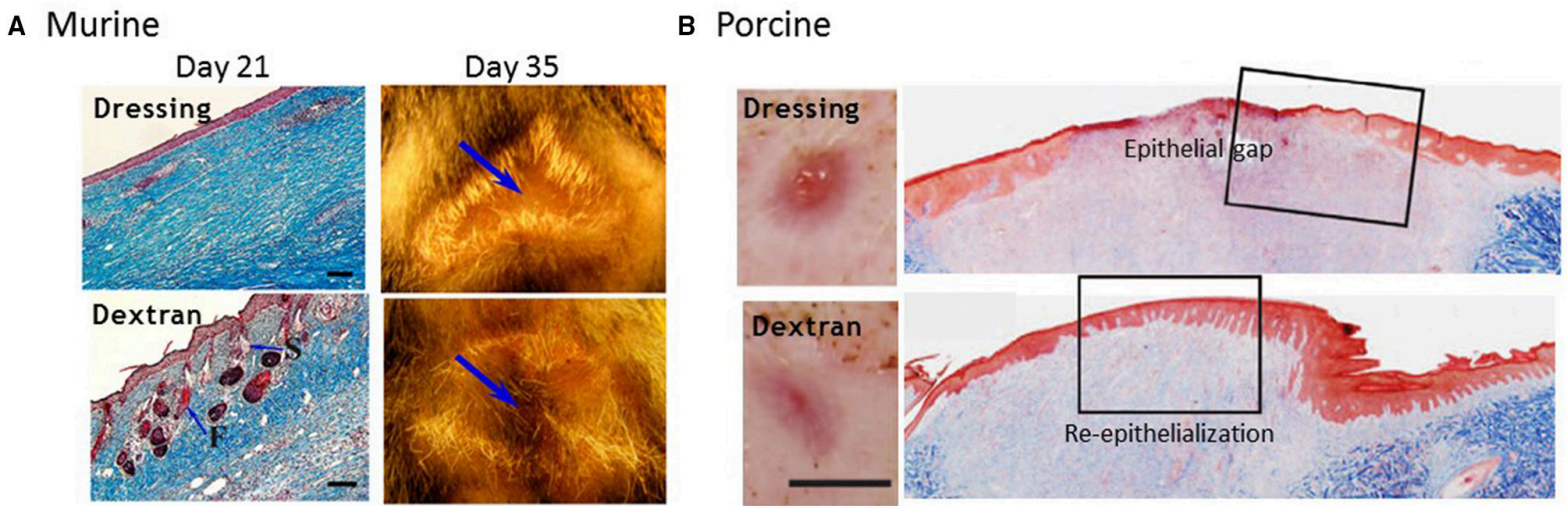
**Biopolymeric dextran scaffold facilitates wound healing in murine and porcine burn models. (A)** Complete healing was observed in mice by day 21. Dextran treated wounds exhibited mature epithelial structures, including hair follicles (F) and sebaceous glands in the dermal layer as indicated by Masson trichrome staining (left panel; scale bar = 100 μm). By day 35, new hair growth was observed in the center of dextran treated wounds, as shown by photos, compared to wounds treated with dressing only; arrows indicate center of wound. **(B)** Wound closure was observed by day 14 in a porcine model as shown by representative macroscopic and immunohistological images. Identification of neoepithelium using Masson's trichrome-stained sections (right panel) confirmed that wounds treated with dressing-only had an epithelial gap, whereas dextran-treated wounds were completely re-epithelialized with a thick reticulated epithelium. Scale bar = 1 cm. Modified from Sun et al. (2011b), and modified and reprinted from Shen et al. (2015). Copyright (2015), with permission from Elsevier.

Among the natural polymers, dextran is a hydrophilic, non-toxic polysaccharide composed of linear a-1,6-linked D-glucopyranose residues with a low fraction of branches extending from α-1,2, α-1,3, and α-1,4 linked side chains. Dextran is synthesized by bacteria, *Leuconostoc mesenteroide*, and is naturally resistant to protein adsorption and cell adhesion, and modification of its polymer backbone allows the development of biomaterials with specific properties. Dextran is also highly water soluble and easily functionalized through its reactive hydroxyl groups. For instance, modifying dextran polymers with polymerizable vinyl groups creates functionalized dextran macromers that present available C = C groups for crosslinking. These modified dextran macromers are then combined with PEG-diacrylate and photopolymerized to produce a hybrid crosslinked scaffold (Sun et al., 2010). The biosynthetic scaffold technology was developed in Dr. Gerecht's laboratory at Johns Hopkins University. The physical properties of the biosynthetic dextran scaffold can be tuned to facilitate cell infiltration and scaffold degradation by modifying the degree of substitution of crosslinking groups and ratio of polymeric components, modified dextran and PEG-diacrylate (Sun et al., 2011a). The degree of substitution, or the number of functionalized hydroxyl groups on the dextran anhydroglucose units, and dextran content combinatorially affect the crosslinking density and, therefore, the porosity, elasticity, and degradation of the scaffold. Tissue ingrowth and regeneration is largely dependent on these physical properties. A reduced degree of substitution of crosslinking groups affects degradation and generates a scaffold with a more porous architecture (~10 μm) that facilitates cell infiltration and migration as well as the diffusion of oxygen and nutrients. Increased dextran content generates a less rigid scaffold but retains structural integrity to enable handling and interface with the wound bed. When applied to 3rd degree burns in murine and porcine models, the dextran scaffold is quickly penetrated, and degraded by early inflammatory cells, promoting the infiltration of necessary cells to re-epithelialize the wound and facilitate skin regeneration. Third degree burns were selected to evaluate the wound healing potential of the dextran scaffold because in preliminary studies, the dextran scaffold demonstrated rapid vascularization when implanted subcutaneously (Sun et al., 2011a). Thermal injuries display increased capillary permeability and thrombosis, so wound healing outcomes are dependent on neovascularization (Rowan et al., 2015). In mice, complete epithelial repair with mature epithelial morphologies was observed, including hair follicles and sebaceous glands, after application of the dextran scaffold, see Figure 1A (Sun et al., 2011b). Accelerated wound closure was also observed in a porcine model after treatment with the dextran scaffold, in which a thick reticulated neoepithelium was regenerated, see Figure 1B (Shen et al., 2015). This is particularly exciting, because the ability to regenerate skin with functional epidermal appendages, such as hair follicles, and sebaceous and sweat glands, has long been and still is a major clinical objective, and challenge, particularly in the healing of chronic wounds in which obtaining wound closure is the primary objective.

## Conclusions

Chronic wounds are characterized by an extremely complex pathophysiology arising from varied etiologies and combined comorbidities including diabetes, immunosuppression, vascular deficiencies, and increased bacterial load that disrupt healing. These wounds suffer from severe molecular and cellular deficiencies and are, unfortunately, heterogeneous across the patient population. This has contributed to the lack of clinical studies directly comparing the efficacy of available products for the treatment of chronic wounds. Performing controlled comparative trials that evaluate the efficacy of advanced wound care products in healing difficult-to-to heal chronic wounds are necessary. Because of the heterogeneity and lack of clinical evidence demonstrating significantly greater performance of specific products, there is currently no single wound dressing or scaffold that is exclusively used for the treatment and healing of all chronic wound types. The treatment paradigm for chronic wounds must shift toward precision medicine strategies that provide personalized therapy based on individual patient need. The development of novel polymers that mimic the ECM and can be modified to incorporate therapeutics, growth factors, antimicrobials, or cells ushers in a new era of customized platform technologies that deliver bioactive components for the treatment of chronic wounds. As chronic wound healing is multifactorial, biopolymeric scaffolds will be designed based on specific patient need to alter the wound bed and provide the optimal wound healing microenvironment. This personalized approach begins with the identification of therapeutic targets and the development of quantitative biomarker assays to allow physicians to stratify patient populations and guide interventional treatments. This may include delivering bioactive VEGF to stimulate vascularization, releasing antimicrobials to control infection, and/or supplying protease inhibitors to mitigate proteolytic activity and stimulate regenerative wound healing.

## Author contributions

LD and SG contributed to writing and revising the manuscript. Both authors approved the final version of this manuscript.

### Conflict of interest statement

Intellectual property related to the biosynthetic dextran scaffold is owned by Johns Hopkins University and licensed to Gemstone Biotherapeutics LLC, of which SG is a cofounder and consultant. SG has a financial interest in Gemstone Biotherapeutics LLC, which is subject to certain restrictions under University policy. The terms of this arrangement are being managed by the Johns Hopkins University in accordance with its conflict of interest policies. Gemstone Biotherapeutics, LLC, partially supported the research work cited in the manuscript; Gemstone Biotherapeutics LLC did not affect the design, interpretation, or reporting of any of the experiments herein. LD is currently an employee of Gemstone Biotherapeutics. All research described in this review of which LD is a contributing author was completed prior to her employment with Gemstone and was therefore conducted in the absence of any commercial or financial relationships that could be construed as a potential conflict of interest. The reviewer JD and handling Editor declared their shared affiliation, and the handling Editor states that the process nevertheless met the standards of a fair and objective review.
